# “Gold Standards,” Plurality and Monocultures: The Need for Diversity in Psychotherapy

**DOI:** 10.3389/fpsyt.2018.00159

**Published:** 2018-04-24

**Authors:** Falk Leichsenring, Allan Abbass, Mark J. Hilsenroth, Patrick Luyten, Thomas Munder, Sven Rabung, Christiane Steinert

**Affiliations:** ^1^Department of Psychosomatics and Psychotherapy, Justus-Liebig-University Giessen, Giessen, Germany; ^2^Department of Psychiatry, Centre for Emotions and Health, Dalhousie University, Halifax, NS, Canada; ^3^The Derner Institute of Advanced Psychological Studies, Adelphi University, Hy Weinberg Center, Garden City, NY, United States; ^4^Faculty of Psychology and Educational Sciences, University of Leuven, Leuven, Belgium; ^5^Research Department of Clinical, Educational and Health Psychology, University College London, London, United Kingdom; ^6^Psychologische Hochschule Berlin, Berlin, Germany; ^7^Department of Psychology, Alpen-Adria-Universität Klagenfurt, Klagenfurt, Austria; ^8^Department of Psychology, Medical School Berlin, Berlin, Germany

**Keywords:** psychotherapy, gold standard, efficacy, evidence, cognitive-behavior therapy

## Abstract

For psychotherapy of mental disorders, presently several approaches are available, such as interpersonal, humanistic, systemic, psychodynamic or cognitive behavior therapy (CBT). Pointing to the available evidence, proponents of CBT claim that CBT is the gold standard. Some authors even argue for an integrated CBT-based form of psychotherapy as the only form of psychotherapy. CBT undoubtedly has its strengths and CBT researchers have to be credited for developing and testing treatments for many mental disorders. A critical review, however, shows that the available evidence for the theoretical foundations of CBT, assumed mechanisms of change, quality of studies, and efficacy is not as robust as some researchers claim. Most important, there is no consistent evidence that CBT is more efficacious than other evidence-based approaches. These findings do not justify regarding CBT as the gold standard psychotherapy. They even provide less justification for the idea that the future of psychotherapy lies in one integrated CBT-based form of psychotherapy as the only type of psychotherapy. For the different psychotherapeutic approaches a growing body of evidence is available. These approaches have their strengths because of differences in their respective focus on interpersonal relationships, affects, cognitions, systemic perspectives, experiential, or unconscious processes. Different approaches may be suitable to different patients and therapists. As generally assumed, progress in research results from openness to new ideas and learning from diverse perspectives. Thus, different forms of evidence-based psychotherapy are required. Plurality is the future of psychotherapy, not a uniform “one fits all” approach.

## Introduction

For psychotherapy of mental disorders, several approaches are available such as interpersonal therapy (IPT), humanistic therapies, cognitive-behavior therapy (CBT), systemic therapy, or psychodynamic therapy (PDT). Whereas some authors argue that at present no form of psychotherapy may claim to be the gold standard [1–3], that is the presently best available treatment, some proponents of CBT explicitly have claimed this prominent status for CBT, both in general ([4], p. 1, 2) and for several mental disorders in particular, such as depressive disorders, anxiety disorders, borderline personality disorder^1^, bulimia nervosa, or post-traumatic stress disorder ([5], p. 879, [6], p. 629, [7], p. 611, [8], p. 679). The American Psychological Association's Division 12 Task Force on Psychological Interventions, for example, which primarily represents CBT researchers, listed CBT as the only treatment with “strong research support” in almost 80% of all the mental disorders included ^2^.

Some authors have recently even argued for developing one “integrated” CBT-based form of psychotherapy as the only type of psychotherapy ([4], p. 1). These authors explicitly argue against pluralism in psychotherapy ([4], p. 1) which is favored by other researchers [3, 9]. This plea against pluralism contradicts the widespread recognized need for plurality of ideas and approaches in science in general, and in the field of psychotherapy in particular [1, 2]. To what extent this rather extreme defense of a monoculture is shared by other representatives of CBT is not clear [10].

Let us be very clear from the outset. CBT has many strengths. Psychotherapy research owes to CBT a systematic and persistent empirical orientation, with regard to developing and testing models and treatments for specific mental disorders. Thus, when we critically discuss the available evidence for CBT in the following, this should not be misunderstood as a kind of “CBT bashing.” It is rather a form of reality testing. What we need in the field of psychotherapy is a constructive yet critical examination of our assumptions and findings, as recently also pointed out by Weisz et al. [11].

## Evidence for CBT

Like psychodynamic therapy or humanistic therapy CBT is an umbrella term including a variety of approaches (behavioral, cognitive, and “third wave” approaches) [4]. Proponents of CBT arguing that CBT is the gold standard refer to the following arguments: (a) more studies are available for CBT than for other psychotherapies, (b) no form of psychotherapy has been shown to be superior to CBT, (c) the theoretical foundations and the (d) mechanisms of change of CBT have been researched most extensively [4].

In the following, we will review these assumptions in the light of empirical evidence. Next, we will discuss the implications of these findings for the future development of psychotherapy.

### Theoretical foundation

Proponents of CBT argue that theoretical foundations and the mechanisms of change of CBT have been most intensively researched [4]. The status of CBT theory, however, may be less solid than assumed by these proponents. Replication efforts of many theories and assumptions in the field of psychology and especially those within the field of cognitive theory, on which CBT is largely based, have widely failed and have led to a crisis in psychology [12]. These findings are highly relevant for CBT. A critical discussion of these findings and of their relevance for CBT, however, is currently missing [4]. Instead, some proponents rather prefer to criticize the theoretical foundations of other approaches ([4], p. 2) which is puzzling.

### Mechanisms of change

CBT puts a specific focus on disorder-related cognitions assumed to maintain mental disorders. For CBT the mechanisms of change are far less clear than claimed by some of its proponents [13]. Alan Kazdin put is as follows ([13], p. 420): “… whatever may be the basis of change with CT [cognitive therapy], changes in cognitions, as originally proposed, are not necessary conditions for therapeutic change.” This result contradicts the cognitive theory of depression [13], a CBT flagship. Furthermore, several studies suggest that CBT involves interventions that actually fall outside of the scope of CBT and that the outcome of CBT is significantly related to these other interventions, such as confrontation and interpretation from psychodynamic models [14–17]. A critical discussion of these results is missing as well [4].

Based on this it is ironic that CBT researchers question the empirical support of other approaches even when they are proved to be superior to CBT—such as panic-focused psychodynamic therapy which was superior to applied relaxation [18]—arguing that the mechanisms of change of this approach have not been empirically studied ([4], p. 2,^2^). Applying the same logic to CBT would reduce the support for CBT considerably since the mechanisms of change are not fully clear for CBT as well. However, none of the CBT proponents seems to have ever applied this argument to CBT, only to other approaches [19].

### Study quality

Some CBT proponents claim that most other psychotherapies “do not even come close” to the quality of studies available for CBT ([4], p. 2). The available evidence once again tells a different story. Several key limitations in this regard have been amply described [2, 20, 21].

(a) Weak comparators: Many studies of CBT have typically used weak comparators [21]. In anxiety disorders, for example, more than 80% of studies used waiting list controls [21]. This is true for depressive disorders in 44% of the studies [21].
(b) High risk of bias: Most CBT studies are judged to be of low quality when the Cochrane risk of bias tool was applied [21]. Only 11 (of 63) studies for major depressive disorder and 21 (of 121) studies for anxiety disorders were found to be of high quality, that is only 17% of studies [21]. However, as this investigation only looked at four of the six Cochrane risk of bias criteria, it is likely that theses numbers of high quality studies are rather optimistic, given that the two missing criteria—selective outcome reporting and blinding of participants and personnel—are known to be important risks of bias in psychotherapy trials. The Cochrane risk of bias tool in its present form, however, may be not an optimal instrument to evaluate psychotherapy studies [22].
(c) Study quality: Study quality of CBT assessed by the RCT-Psychotherapy Quality Rating Scale (RCT-PQRS) proved not to be superior to that of other approaches such as PDT [23].
(d) Insufficient power: Proponents of CBT argue that CBT was tested in studies using “the most stringent research criteria,” for example an active comparator ([4], p. 2). In fact, however, as reported above, CBT in anxiety disorders, for example, was mostly (80%) tested against a waiting list comparator [21]. Comparisons against active comparators exist, too; however, none of the studies comparing CBT to an active comparator was sufficiently powered [20]. These studies do not allow for definite conclusions in case of non-significant differences between treatments [24].
(e) Allegiance bias: Researcher allegiance has been found to have a major impact on results of comparative psychotherapy outcome research [25–27]. For this reason, it was called a “wild card” in psychotherapy outcome research ([25], p. 95). At present, however, researcher allegiance is not yet sufficiently controlled for [22], for example, by the Consolidated Standards of Reporting Trials (CONSORT, [28]) or the Template for Intervention Description and Replication (TIDieR, [29]). For several studies of CBT high risk for a researcher allegiance in favor of CBT was demonstrated [1, 2, 22], and for most CBT studies a high-risk of bias [21].
(f) High uncertainty: Due to the low number of high quality studies and the large number of studies with a high risk of bias the authors of a large meta-analysis on depressive and anxiety disorders concluded that the effects of CBT are “uncertain and should be considered with caution” ([21], p. 245). They regard CBT as only “probably effective” ([21], p. 245).

### Quantity does not imply quality

CBT proponents claim that for CBT much more studies exist than for most other psychotherapies [4, 30]. This is true, probably also for high-quality studies, but for other approaches there is a growing number of studies [31–36]. Furthermore, as shown above, quantity should not be confused with quality, especially in terms of efficacy. More studies do not imply higher efficacy. Since differences in efficacy between bona fide psychotherapies are generally found to be small [1, 2, 37], it is unlikely that in high-quality studies substantial differences in favor of CBT will emerge. In a recent meta-analysis testing equivalence, for example, differences between CBT and PDT in target problems were small and clinically not important (*g* = 0.16 post-therapy, 0.05 at follow-up) [33].

### Efficacy I: CBT is not a panacea

For a treatment claiming to be the gold standard the question of efficacy is crucial. The efficacy of CBT, however, is less strong than usually assumed.

(a) Limited superiority to controls: As a surprising result, some meta-analyses found CBT not to be superior to psychological placebo in depression [38] or to control conditions in borderline personality disorder [39]. In high-quality studies of depressive disorders, CBT achieved only a small effect size (*d* = 0.22) compared to treatment as usual (TAU) or placebo [40]. This effect size is below the threshold of 0.24 suggested as a clinically minimally important difference in depressive disorders [41]. Furthermore, CBT was found to be ineffective in reducing symptoms of schizophrenia and relapse in bipolar disorder [42, 43]. Bias significantly affected effect sizes with *g* = 0.15 in masked vs. *g* = 0.62 in non-masked studies [43]. Thus, the additional gain of CBT over TAU seems to be limited. This may be true for other psychotherapies as well, but they do not claim to be the gold standard.
(b) Limited response and remission rates: Significant mean differences are of limited value for judging the benefit of a treatment in individual patients. In this respect, rates of response and remission are more informative. Success rates achieved by CBT are typically modest. Across anxiety disorders, for example, a mean response rate of 49.5% was reported ([44], p. 76). Similar results were found for major depressive disorder (53%) ([45. p. 121). Rates for remission are even smaller, often about 25% [46]. Thus, a considerable proportion of patients does not sufficiently benefit from CBT. Apparently, CBT is not a panacea. There is clearly room for improvement, and this is true for all currently tested bona fide treatments.

### Efficacy II: CBT lacks superiority to other treatments

A gold standard treatment can be expected to be clearly superior to other treatments.

(a) No evidence for superiority: There is no consistent evidence that CBT is superior to other treatments [1, 2]. When differences were found in some studies, effect sizes were small and negligible [1, 2, 22], often related to highly specific measures tailored to CBT (e.g. of cognitions) as opposed to broad band functioning [1]. At least in some studies, these small differences may be due to researcher allegiance effects [1, 2, 22]. Furthermore clinicians vary in their efficacy, both within and between treatment conditions, not only in psychotherapy, but also when delivering pharmacotherapy ([2], p. 170, [47]). Failure to take therapist effects into account may result in increased type I errors and overestimating treatment effects, for example erroneously concluding “treatment A is superior to B” (2, p. 164, [48]). This problem, however, is not specific to CBT.

Some CBT researchers claim that even small differences may be clinically relevant, without, however, providing evidence specifically for CBT [4, 49]. In depression, for example, differences in efficacy (*d* = 0.16) for comparing CBT with other psychotherapies do not even exceed the proposed threshold for a clinically minimally important difference (*d* = 0.24) [37]. This is true for anxiety disorders (*d* = 0.14) and PTSD (*d* = 0.19) as well [49, 50]. In general, differences between bona fide psychotherapies are small (about *d* = 0.20) [51]. This is true for specific psychotherapies such as interpersonal therapy (IPT) and PDT when compared to other psychotherapies, for example in depressive disorders (IPT: *g* = 0.06, PDT: *d* = −0.14) [52, 53] or for PDT across mental disorders (*g* = −0.10) [54].

These results from randomized clinical trials are consistent with those of recent large-scale naturalistic studies in the United Kingdom [55]. These findings have, for instance, inspired the UK government to offer patients a choice between different empirically supported treatments.

(b) Evidence for equivalence: Requiring relatively large sample sizes, only a few studies in psychotherapy research are sufficiently powered to demonstrate equivalence or non-inferiority [20, 24]. The lack of statistical power can be solved by use of meta-analysis [56]. For testing equivalence, a margin has to be specified that is regarded as compatible with equivalence [57]. In addition, the efficacy of the active comparator must be ensured [19, 58]. A recent equivalence meta-analysis fulfilled these requirements [33]. In addition, researcher allegiance was explicitly controlled for, both on an experimental and a statistical level [33]. This meta-analysis found PDT to be as efficacious as treatments established in efficacy including CBT [33]. The results of this meta-analysis, however, were recently misinterpreted by a number of CBT proponents as favoring CBT, by stating that ([4], p. 2): “… in the equivalence limit, significant differences…were found.” Apparently, the authors have misunderstood the logic of equivalence testing. In equivalence testing a statistically significant result implies that the effect size and its CI is within the equivalence margin, thus demonstrating equivalence [57].
(c) Specific factors in psychotherapy: Another line of research suggests that the variance explained by therapy-specific factors (techniques) is rather limited [1, 2, 59]. Meta-analyses did not find evidence that specific factors contribute significantly to treatment outcome [60, 61]. These results generally question that one form of therapy may be the gold standard, that is the best available form of psychotherapy.

### Does the evidence suggest a CBT-centered monoculture or a plurality of approaches?

While other researchers plead for plurality in research and treatment [1–3], some proponents of CBT argue for developing an integrated CBT-based form of psychotherapy as a general and apparently the only form of psychotherapy ([4], p. 2): “While many non-CBT psychotherapies have changed little in practice since their creation…continuous improvements in psychotherapy will derive from CBT, gradually moving the field toward an integrative scientific psychotherapy,” “… with CBT serving as as the foundational platform for integration” ([4], p. 1). This claim presents a seriously distorted picture of other treatment approaches [9], again raising the issue of researcher allegiance. Furthermore, it is not consistent with the available evidence, as shown above and further elaborated in the following.

(a) No improvement during recent decades: In fact, outcome of CBT does not seem to have improved over the course of time. During the recent 40 years, effect sizes for CBT in anxiety disorders were found to have stagnated [62], for depressive disorders even a significant decline was reported [63]. Also for psychotherapy in youth, including CBT, there has been no increase in efficacy across five decades of research [11]. Thus, CBT did not show the claimed “continuous improvement” ([4], p. 1). Whether other psychotherapies showed such an improvement, however, is not known. Nevertheless, the results available for CBT suggest that not only or primarily research in CBT should be carried out and funded - which seems to be the case (see below) ^3^.
(b) Progress in other psychotherapies: There is no evidence for the assertion that little change was achieved in other approaches. In the area of PDT, IPT, or humanistic therapy, for example, efficacious disorder-specific and manualized treatments have been developed [31, 32, 64, 65]. Often, however, a distorted picture of other approaches such as PDT is publicly presented [9], especially by some CBT proponents [4]^2^.
(c) Incorporating elements of other approaches: Claiming that improvements in psychotherapy will derive from CBT apparently denies that CBT benefited from integrating concepts of other therapeutic approaches [64], often under a new name, frequently without citing their origins. The unified protocol by Barlow et al. for example addresses defense mechanisms under the newly coined term of “emotional driven behaviors and emotional avoidance,” with no reference to psychodynamic therapy ([66], p. 886, [67]). Schema-focused therapy integrated concepts of PDT and humanistic-experiential therapies, too, but cited these origins [68]. This is also true for the Cognitive Behavioral Analysis System of Psychotherapy (CBASP) [34]. Thus, many innovations of CBT stem from other approaches and, thus, are actually examples for the benefits of pluralism in psychotherapy.
(d) The tendency to incorporate elements of other approaches such as PDT is the more puzzling since, on the other hand, psychodynamic concepts have often been criticized by CBT researchers as “unsupported” ([4], p. 2)^2^. So why include them in CBT?—Take them, rename them, and devalue its origin?
(e) Clinical practice: In clinical practice, many therapists apply non-CBT approaches such as psychodynamic, integrative or humanistic [69]. Not even one quarter reported to use CBT [69]. Do authors like David et al. [4] suggest that all of these therapists “convert” to CBT if only a CBT-oriented general psychotherapy remains? The evidence does not support this.

## Conclusions: the future

At present, no form of psychotherapy may claim to be the gold standard. Rather many can claim to be beneficial, and none without limitation. For CBT the evidence is less robust than often portrayed. It is of note that many of the reviewed results and conclusions pointing to limitations of CBT were reported by CBT-oriented or independent researchers [10, 13, 20, 21, 23, 38, 39, 44–46, 49, 62, 64]. Thus, these results and conclusions cannot be simply attributed to a bias against CBT.

With the limitations listed above, there is evidence for CBT. This is true for other approaches as well, including psychodynamic therapy, interpersonal therapy, humanistic therapy and systemic therapy [31–36]. In Germany, for example, psychodynamic therapy, systemic therapy, and CBT have been certified as scientific and efficacious treatments by the scientific board of psychotherapy (wbpsychotherapie.de), the paramount board to evaluate the scientific status of psychotherapies. It is of note that in this board, proponents of CBT and PDT are represented on equal terms.

With response rates of 50% or below and remission rates that are even lower, CBT is not a panacea. This is true for other approaches as well [45]. For this reason, a plurality of research-supported approaches may be advantageous, for example, in patients not responding to one therapy approach. In contrast, a plea for a “scientific” integrated psychotherapy under the hegemony of CBT [4] implies that other approaches are not scientific: this, itself is a non-scientific position. Other prominent CBT researchers, however, do not seem to share this CBT-centered view ([10], p. 33): “If the question at hand is whether research is far enough along to support the view that only CBTs should be investigated, taught in training programs, and offered to individuals with mental health problems, then the answer is clearly ‘no'.”

Thus, there is a need for studying not only CBT, but other approaches as well. Open questions need to be addressed. At present, for example, it is widely unclear what patients benefit from which approach. Do patients who did not respond to one approach benefit from another, for example non-responders of PDT from CBT and vice versa? Whereas shifting from one treatment to another is common in pharmacotherapy, it has hardly been studied in psychotherapy [70]. In addition, further research on dose-effectiveness relations is required, especially for patients with chronic disorders or personality disorders for whom short-term treatments seem not to be sufficient [71].

Studies addressing these issues need to be supported by funding organizations—in contrast to pharmacotherapy, there is no industry funding research in psychotherapy. Funding organizations, however, were shown to prefer mainstream research [72]. Many review, funding and guideline committees are largely dominated by CBT researchers which poses another threat in terms of allegiance bias and hampers research in other approaches [9]. A bias in funding is demonstrated, for example, by data from the UK^3^: With 1.96% of total research funding, psychodynamic therapy, for example, is one of the least well-funded psychotherapies, compared to 40.6% of funding for variants of CBT (cognitive therapy, behavior therapy, behavior activation, mindfulness therapy, acceptance and commitment therapy, problem solving therapy, cognitive remediation therapy, and exposure). Here, CBT is in fact the “gold” standard. In the US or Germany, data are likely to be similar^4^. Considering these differences in funding, it is actually quite surprising that evidence for CBT is where it is. Anyway, a change in funding policy is urgently required.

In addition, also non-CBT-approaches need to be taught at the universities and in training institutions. In some countries such as Germany, 99% of chairs in clinical psychology and psychotherapy are held by representatives of CBT—a situation that can hardly be justified by the evidence reported above. Only if other psychotherapies are taught and studied, they will be able to further contribute to the development of psychotherapy at large.

The different psychotherapeutic approaches have their strengths, be it a focus on interpersonal relationships, on cognitions and learning, on experiential, affective or unconscious and defensive processes. Different patients may benefit from different approaches, or may benefit through different routes. Therapists are different as well. They should be able to choose which approach fits them best: One size does not fit all. Also learning from each others' approaches requires that different forms of evidence-based psychotherapy exist and are valued equally [64]. Plurality is the future of psychotherapy, not a CBT-centered “one fits all” monoculture.

## Author contributions

All authors listed have made a substantial, direct, and intellectual contribution to the work, and approved it for publication.

^4^

### Conflict of interest statement

FL, AA, MH, PL, CS research, teach, and practice psychodynamic therapy (PDT) and have published books or book chapters dealing with PDT. SR has published books or book chapters dealing with PDT. FL has been trained in PDT, CBT and client-centered therapy. SR has been trained in CBT. AA, MH, PL, TM, CS have been trained in PDT. All authors trained scientists, active promotors, and contributors to evidence-based psychotherapy.

## Footnotes

^1^Linehan Institute: http://behavioraltech.org/resources/whatisdbt.cfm

^2^www.div12.org/psychological-treatments/

^3^MQ: MQ Landscape Analysis. UK Mental Health Research Funding (2015)

^4^Funding organizations like the German Research Foundation (DFG) are reluctant to publish data for the different psychotherapeutic approaches on rates of grant applications compared to actual funding. Here, more transparency is definitely required.
